# Supplementary material for: Bile Acids as Emerging Players at the Intersection of Steatotic Liver Disease and Cardiovascular Diseases
Source: Biomolecules. 2024 Jul 12;14(7):841. doi: 10.3390/biom14070841 (PMC11275019; doi:10.3390/biom14070841)
Supplement: Supplementary file 1 [file biomolecules-14-00841-s001.zip › biomolecules-3107810-supplementary.pdf]

Supplementary material

Table S1. Summary of publications exploring relationship between circulating bile acids and MASLD severity.

Study	Method of assessment and population groups	Method of BA analysis	Key observation	Adjusted for potential confounding factors?
Bechmann et al. (2013)[1]	**Liver biopsy** Patients with NASH (n=98) Without NAFLD (n=10)	Enzymatic assay	Total serum BA concentration increased with increasing NAFLD activity score (Rho: 0.47, *P*<0.001) and were higher in patients with NAFLD with histological evidence of 'many' compared to 'few' ballooned hepatocytes (approx. 2-fold increase). A noticeable degree of variant in total BA concentrations within patients with NAFLD (~0.5 - 50.0 mg/dl)	No
Ferslew et al. (2015)[2]	**Liver biopsy** Patients with NASH (n=7) Healthy controls (n=15)	LC/MS	Fasting concentrations of total BAs in patients with NASH were increased 2.2 to 2.4-fold compared to healthy participants (P<0.05). Taurine- and glycine-conjugated BAs were 5.6-fold and 3.2-fold times higher in patients with NASH compared to healthy participants.<br><br>Concentrations (mean ± SEM) of **3-DHCA** (1.2 ± 0.2 vs 3.0 ± 0.8), **GCA** (45.6 ± 17.8 vs 186.0 ± 62.3), **GCDCA** (243.0 ± 45.3 vs 694.0 ± 189.0), **GLCA** (10.3 ± 3.2 vs 31.4 ± 11.0), **TDCA** (20.5 ± 6.6 vs 124.0 ± 67.6), **TUDCA** (1.5 ± 0.3 vs 4.3 ± 1.4 nM) were lower in healthy controls vs patients with NASH.	No
Jahnel et al. (2015)[3]	**Liver biopsy** Children with NAFLD (n=92) Healthy controls (n=105)	HPLC-MS/MS	Total BA concentrations (mean ± SD) were lower in children with NAFLD without fibrosis (1.7 ± 1.2) or with fibrosis (2.5 ± 1.8) compared to healthy controls (3.6 ± 2.0 µmol/L). Glycine- and taurine-conjugated BAs were lower in children with NAFLD compared to healthy controls (both P<0.05).	No
Jiao et al. (2018)[4]	**Liver biopsy**	LC/MS/MS	Total serum BA concentrations 3-fold times higher in patients with	No

	Patients with biopsy proven NASH (n=16)<br>Healthy controls (n=11)		NASH compared to healthy controls.<br>The percent quantity of **DCA** in total BAs was ~4 times higher (absolute concentration in control group 0.2 µM)) in patients with NASH compared to healthy controls.	
Puri et al. (2018)[5]	**Liver biopsy**<br>Patients with NAFL (n=25)<br>Patients with NASH (n=37)<br>Healthy controls (n=24)	LC/MS	There was a step wise increase of total primary BAs from control to NAFL to NASH (P<0.05 NASH vs control) whilst total secondary BAs were decreased with increasing disease severity. Patients with NASH had significantly increased plasma **GCA** and **TCA**, p<0.05; **GCDCA** and **TCDCA**, p<0.05; and **GUDCA** and **TUDCA**, p<0.05 compared to healthy controls when BA concentrations were expressed relative to library standards across samples.	No
Chen et al. (2019)[6]	**Liver biopsy**<br>Patients with NAFLD (n=72)<br>Healthy controls (n=15)	LC/MS	Total primary BAs increased (38.9 vs 54.4% in healthy controls vs patients with NASH respectively) whilst total secondary BAs (61.1 vs 45.6% in healthy controls vs patients with NASH) decreased with disease severity.<br><br>The relative presence of **TCDCA** (1.3 vs 3.2%) and **GCDCA** (18.6 vs 28.1%) were the most increased in patients with NASH compared to healthy controls (both P<0.05). The relative presence of **MCA** BAs was the most noticeably decreased secondary BAs (0.2 vs 0.1%) in patients with NASH compared to healthy controls, however, these represented a very small proportion of total BAs.	No
Yara et al. (2019)[7]	**Liver biopsy**<br>Patients with NAFLD (n=34)<br>Healthy controls (n=38)	LC/MS-MS	The mean total serum bile acid concentration in patients with NASH (8.2 ± 1.0 µmol/L) was significantly higher (*P* <0.0001) compared with healthy controls (4.0 ± 0.3 µmol/L), and this higher level in patients with NASH did not depend on the progression of hepatic fibrosis (<F2 vs ≥F2: 8.3 ± 1.4 µmol/L vs 8.0 ± 1.4 µmol/L).	No

			Taurine- (~3 fold higher) and glycine-conjugated (~3.5 fold higher) BA fractions were higher in patients with NASH compared to healthy controls (both P<0.01).	
Caussy et al. (2019)[8]	**MRI-PDFF**<br>Patients with NAFLD (n=36)<br>Participants without NAFLD (n=120)	LC/MS	No difference in total BA concentrations between groups (*P*=0.56). Median (IQR) relative presence (%) of CA; 4.6 (16.5) vs 3.7 (14.5), conjugated **CDCA** BAs; 9.0 (5.5) vs 6.5 (4.3) were higher whilst GHCA; 1.2 (2.0) vs 3.6 (3.9) was lower in patients with NAFLD compared to healthy controls (all P<0.05). Increased proportion of GHCA remained associated with a lower risk of NAFLD (OR 0.76; 95%CI: 0.57-1.00) independently specific confounding factors.	Yes (age, sex, Hispanic ethnicity, T2DM and obesity).
Zhang et al. (2020)[9]	**Ultrasonography**<br>Participants without NAFLD by ultrasonography (n=110,565)<br>Participants with NAFLD by ultrasonography (n=41,771).	Enzymatic assay	Median (Q1-3 quartiles) concentrations of **total serum BAs** were slightly higher in those with; 3.4 (2.3-5.4) vs without NAFLD 3.0 (1.9-4.8) µmol/L. **Total BA concentrations** were not associated with NAFLD (OR= 1.00 (95%CI; 1.00-1.00) P=0.80) after adjusting for age, sex, BMI, and other risk factors for NAFLD.	Yes (age, sex BMI, hypertension, T2DM, total cholesterol, LDL-C, HDL-C and triglyceride).
Adams et al. (2020)[10]	**Liver biopsy**<br>Patients with NAFLD with no-mild fibrosis (n=58)<br>Patients with NAFLD with F3-4 fibrosis<br>Patients with NAFLD with advanced liver fibrosis (n=9)<br>Participants without NAFLD (n=55)	LC/MS-MS	Median (IQR) **total BA** concentrations were higher in patients with NAFLD (F0-2 fibrosis) 1.01 (0.40 -2.72) compared to healthy controls 0.54 (0.23-1.08) and were further elevated in patients with NAFLD (F3-4 fibrosis) 2.12 (1.44-5.73) µM. Concentrations of **primary and secondary conjugated BAs** were both higher in patients with NAFLD (F3-4) compared to healthy controls (Total primary conjugated BAs; 0.19 vs 1.44, Secondary conjugated BAs; 0.21 vs 0.83, both P<0.001). Following adjustment for clinical factors also associated with advanced fibrosis, only primary (including **GCA** and **GCDCA**]) and secondary conjugated BAs	Yes (age, sex BMI, hypertension, T2DM, total cholesterol, HOMA-IR, HDL-C, and triglyceride).

			(including **GDCA**) remained significant.	
Sydor et al. (2020)[11]	**Ultrasound or liver biopsy**<br>Patients with NASH without HCC and without (n=23) or with (n=11) cirrhosis. Patients with NASH with HCC and without (n=14) or with n=19).<br><br>Healthy controls (n=20)	HPLC-MS/MS	Compared to healthy controls, concentrations of total BAs were approximately 10-fold higher in patients with NASH and cirrhosis both without and with HCC. **Primary conjugated BA** concentrations were higher in patients with NASH and cirrhosis (both with and without HCC) compared to healthy controls although the effect size for this difference is not clear.	No
Nimer at al. (2021)[12]	**Liver biopsy**<br>Patients with biopsy proven NAFLD (n=102)<br>Healthy controls (n=50)	LC/MS/MS	Compared to healthy controls, almost all concentrations of BAs were elevated in patients with NAFLD besides from LCA3-Sulfate. Plasma 7-Keto-DCA concentrations showed the strongest associations with advanced stages of hepatic fibrosis [odds ratio(95%CI)], 4.2(1.2–16.4), NASH 24.5(4.1–473.0), and ballooning 18.7(4.8–91.9).	No
Chen et al. (2021)[13]	**Liver biopsy**<br>Patients with NAFLD (n=87)<br>Healthy controls (n=35)	LC-MS/MS	Total BA concentrations were higher in patients with NAFL (13.6 ± 14.0) or NASH (15.8 ± 25.4) compared to healthy controls (8.3 ± 5.9). Ratios of GCA/TCA, GDCA/TCDCA, GDCA/TDCA were all higher in patients with NAFL compared to healthy controls.	No
Jung et al. (2021)[14]	**Liver biopsy**<br>Patients with NAFL (n=99)<br>Patients with NASH (n=75)<br>Participants without NAFLD (n=67)	UPLC/Q TOF-MS	Total BA concentrations were increased in patients with NASH compared to NAFL (Approx 1.3-fold increase. ~4,000 vs ~6,000 nM in NAFL and NASH group respectively, P=0.03).	No - differences remained after stratification by T2DM status.
Wu et al. (2021)[15]	**Ultrasonography**<br>Patients with T2DM with NAFL (n=30)<br>Patients with T2DM without NAFL (n=36)	UPLC/TQ-MS	Total taurine-taurine conjugated BAs were higher in those with both T2DM and NAFL (191.2 (107.8-394.2)) compared to those only with T2DM (81.7 (38.8-176.9) nmol/L). Concentrations of **TCA**, **TCDCA**, **TDCA**, **TUDCA**, **LCA**, **TLCA**,	Yes - age, sex, BMI, WHR, FPG, 2h PG, ALT, Triglyceride, HDL-C, and LDL-C

			**CDCA-24G** and **HDCA** were all at least 2-fold higher in patients with both T2DM and NAFL compared to those with only T2DM (all P<0.05).	
Xie et al. (2021)[16]	**Liver biopsy**<br>Patients with NASH and fibrosis (n=99)<br>Healthy controls (n=99)	UPLC/MS	The concentrations of all 15 analysed BAs were significantly higher in patients with NASH and liver fibrosis compared to healthy controls (all but two P<0.001). Relative to healthy controls, the concentrations of **12α-OH BAs** were approximately 14-fold times higher in patients with NASH and fibrosis (healthy control concentrations approx. 200 ng/ml). The increase in **12α-OH BAs** in patients with NASH and fibrosis compared to healthy controls was noticeably greater than the increase in non-**12α-OH BAs** (P=0.0001).	No
Kasai et al. (2022)[17]	**Liver biopsy**<br>Patients with NAFLD with mild fibrosis (n=104)<br>Patients with NAFLD and advanced fibrosis (n=95)<br>Healthy controls (n=55)	LC-MS/MS	**Total CA** concentrations were higher in patients with NAFLD, and mild fibrosis (1.18 ± 0.89) compared to healthy controls (0.44 ± 0.38) and were even higher in patients with NAFLD and advanced fibrosis (1.94 ± 1.43) (differences between all groups P<0.001). Simiarly, **total LCA** concentrations were highest in the NAFLD advanced fibrosis group (1.45 ± 0.71) compared to the NAFLD mild fibrosis (1.03 ± 0.49) and healthy control (0.66 ± 0.27) groups (P<0.001). Differences in total LCA concentrations were predominantly driven by increased **unconjugated LCA** BAs in those groups with more severe liver fibrosis.	Yes – BMI and HOMA-IR
Rivera-Andrade (2023)[18]	**Fatty liver index**<br>Patients with NAFLD (n=251)<br>Participants without NAFLD (n=164)	LC-MS/MS	Compared to those with no NAFLD, patients with NAFLD had higher median (IQR) concentrations of **total primary bile acids** (498.0 (212.5-967.8) vs 575.4 (288.5-1,048.1) ng/ml), P=0.05), and **total secondary bile acids** (83.0 (32.6-164.5) vs 102 (49.8-220.0) ng/ml),	No

			P=0.01).There was no significant difference between the two groups in the ratio of **secondary-to-primary bile acids**. Patients with NAFLD had significantly higher median concentrations of **GCA** (p=0.002), **TCA** (p<0.001), **TCDCA** (p<0.001), **DCA** (p=0.01), **GDCA** (p=0.04), and **TDCA** (p=0.002).	
Liu et al. (2023)[19]	**Liver biopsy** Patients with NAFLD without (n=145) with liver fibrosis (F1-F4; n=405)	UPLC-MS/MS	Patients with NAFLD and mild fibrosis had higher concentrations of secondary BAs compared to patients with NAFLD without liver fibrosis although effect size was unclear.	No
Fitzinger (2024)[20]	**Ultrasound and ELF** Patients with NAFLD (n=45) Healthy controls (n=103)	LC/HR-MS	Compared to healthy controls, female patients with NAFLD had significantly increased total (2.89 (1.40, 6.50) vs. 1.18 (0.86, 2.30), p = 0.009) and total primary BA (1.62 (1.25, 3.45) vs. 0.65 (0.45, 1.11), p = 0.003) concentrations. Women with NAFLD also showed significantly increased total **CA** (0.46 (0.21, 0.94) vs. 0.16 (0.08, 0.33), p = 0.003), total **CDCA** (1.16 (0.68, 2.88) vs. 0.46 (0.33, 0.77), p = 0.004), total glycine-conjugated BA (1.89 (1.04, 5.15) vs. 0.65 (0.40, 1.10), p = 0.007), and total non-12-OH BA (1.45 (0.80, 3.86) vs. 0.61 (0.45, 1.07) p = 0.010). Concerning BA subfractions, **GCA** (0.19 (0.12, 0.68) vs. 0.07 (0.04, 0.12), p < 0.001), and **GCDCA** (0.84 (0.61, 2.27) vs. 0.25 (0.17, 0.44), p = 0.005,) were higher in women with NAFLD.<br>Compared to healthy controls, male patients with NAFLD had no significantly increased total BA. Nevertheless, the BA subfractions **GCA** (0.20 (0.09, 0.35) vs. 0.08 (0.05, 0.16), p < 0.001), **GCDA** (0.30 (0.24, 1.11) vs. 0.18 (0.10, 0.41), p = 0.003), **LCA** (0.006 (0.001, 0.019) vs. 0.001 (0.001, 0.001), p < 0.001), **TLCA** (0.0010 (0.0010, 0.0023) vs. 0.0010 (0.0010, 0.0010), p < 0.001), and **TDCA** (0.0010 (0.0010, 0.009) vs. 0.0010 (0.0010, 0.0010), p < 0.001) were significantly increased.	No

			**CA** (0.04 (0.01, 0.12) vs. 0.20 (0.17, 0.28), p < 0.001) was significantly decreased.	

Abbreviations: NASH; non-alcoholic steatohepatitis, NAFLD; non-alcoholic fatty liver disease, BA; Bile acid, LC; liquid chromatography, MS; mass spectrophotometry, 3-DHCA; 3α,7α-dihydroxycholestanoic acid, GCA; glycocholic acid, GCDCA; glycochenodeoxycholic acid, GLCA; glycolithocholic acid, TDCA; taurodeoxycholic acid, TUDCA; tauroursodeoxycholic acid, HPLC; high-performace liquid chromatography, DCA; deoxycholic acid, TCDCA; taurochenodeoxycholic acid, GUDCA; glycoursodeoxycholic acid, MCA; muricholic acid, CDCA; chenodeoxycholic acid, GDCA; glycodeoxycholic acid, TCA; taurocholic acid, T2DM; type 2 diabetes, LDL-C; low-density lipoprotein cholesterol, HDL-C; high-density lipoprotein cholesterol, LCA; lithocholic acid, CDCA-24G; chenodeoxycholic Acid 24-Acyl-β-D-Glucuronide, HDCA; Hyodeoxycholic acid, 12α-OH; 12α-hydroxylated, LCA; lithocholic acid, CA; cholic acid, TLCA taurolithocholic acid.

Table S2 – Summary of publications exploring relationship between bile acids and cardiovascular diseases.

Study	Method of assessment and population groups	Method of BA analysis	Key observation	Adjusted for potential confounding factors?
Steiner et al. (2011)	**Coronary angiography for the evaluation of established or suspected CAD**<br><br>75 patients with CAD<br>74 patients without significant CAD	HPLC	No detected differences in any of the 15 quantified BAs	No
Mayerhofer et al. (2017)	142 patients with heart failure for <6 months<br><br>20 sex- and age-matched healthy control participants	LC-MS/MS	Plasma levels of primary BAs were lower in patients with heart failure 1.70 (0.72, 3.74) compared to healthy control participants 3.98 (1.98, 5.71) µM ($P < 0.01$). Differences were primarily driven by lower concentrations of CDC, GCDC, TCDC and GCA in patients with heart failure. Conversely, concentrations of multiple secondary BAs (namely GUDC, GLC and TLC) were higher in those with heart failure compared to healthy controls. The ratio of primary and secondary BAs was significantly different between participants with compared to those without heart failure.	eGFR and current smoking status
Zhang et al. (2019)	**Coronary computed angiographic observations**<br><br>101 high-risk coronary plaque subjects	TBA measured by enzymatic assay	Subjects with high-risk coronary artery plaques [6.18 (IQR 5.29, 7.30) µmol/L] had higher serum total bile acid level than in the controls [3.16 (IQR 2.18, 4.01) µmol/L] ($P<0.001$). The area under	BMI, fasting glucose, TG

	93 non-high-risk coronary plaque subjects		the ROC curve (AUC) of serum total bile acid level for predicting high-risk coronary plaques among populations was 0.876 (95% CI: 0.821–0.919; P<0.001), with a sensitivity of 87.13% and a specificity of 86.02%	
Li et al. (2020) [21]	**CAD was diagnosed based on the presence of at least 50% coronary stenosis in at least one major coronary artery according to the CAG results assessed by at least two experienced interventional cardiologists.**<br><br>1,585 non-CAD<br>5,853 CAD	TBA measured by enzymatic assay	Serum TBA of patients with CAD was lower than in patients without CAD (3.4 (2–5.7) vs 4 (2.4–6.5) µmol/L).<br>TBA had an L-shaped association with the presence of CAD and MI and the severity of coronary lesions.	Sex, age, smoker, hypertension, diabetes, heart rate, TC, TG, HDL, LDL, hs-cTnT, NT-proBNP, HbA1c, ALT, ALB, eGFR, use of aspirin, use of P2Y12 inhibitors and use of statins.
Feng et al. (2021)[22]	**CAD was assessed based on coronary angiography (lesions narrowing more than 50% defined as CAD). CAD were also diagnosed as MI based on medial history and related examinations.**<br><br>7,616 post-menopausal women without CAD<br><br>12,639 post-menopausal women with CAD	TBA measured by enzymatic assay	Both in the general population and in the T2DM subgroup, the TBA level was significantly lower in CAD patients than in non-CAD patients. Multivariate regression analysis suggested that low TBA level was positively associated with the occurrence of CAD combined with T2DM (OR: 1.451; 95%CI: 1.141–1.847).	Age, BMI, SBP, DBP, LDL-C and medical history of T2DM, hypertension, hyperlipidemia, smoking, and familial CVD
Chong et al. (2021) [23]	**Coronary computed angiographic observations**<br><br>35 non-CAD<br>45 CAD	HPLC-MS/MS	TBA concentration was two-fold lower in patients with than without CAD. Primary, secondary, and conjugated BA concentrations were all decreased in patients with than in patients without CAD. No observed differences in serum measurements of 7alpha	Age and sex

			C4 (0.13 ± 0.01 vs 0.12 ± 0.01 μmol/l)	
**Liu et al. (2023)** [24]	**Hospitalized patients diagnosed with acute coronary syndrome (ACS)**	TBA measured by enzymatic assay	Logistic regressive analysis disclosed that serum TBA concentrations was an independent predictor of coronary lesions (high vs. low SS: serum TBA adjusted odds ratio (aOR) = 0.8, 95% confidence interval (CI): 0.6–0.9, p < .01.	Age, CK, CKMB, cTNT and CRP
**Wen et al. (2024)** [25]	**Composite endpoints, including all-cause death, cardiac death, unstable angina (UA), nonfatal MI, urgent coronary revascularization (included stent thrombosis and in-stent restenosis), heart failure, and cerebrovascular events (included TIA, ischemic stroke or cerebral bleeding), were defined as MACEs**<br><br>425 patients diagnosed with acute coronary syndrome and following a percutaneous coronary intervention followed for two-years.	TBA measured by enzymatic assay	The areas under the ROC curve of baseline serum TBA levels predicted MACEs in ACS and ACS-T2DM patients following PCI were 0.649 (95% CI 0.595–0.703) and 0.783 (95% CI 0.685–0.881), respectively. Furthermore, Cox regression analysis showed that baseline serum TBA level was associated with the occurrence of MACEs in patients with ACS after PCI over a 2-year follow-up period (HR 1.03, 95%CI; 1.01-1.05, P<0.001).	Age, Sex, Hypertension, T2DM, NYHA classification, systolic and diastolic pressure, Past PCI or CABG, LDL0C, ALT, CKMB and pro-BNP
**Mateu-Fabregat et al. (2024)** [26]	**Participants underwent a coronary angiography. ACS was defined as patients with ST-segment elevation myocardial infarction (STEMI), non-ST segment elevation myocardial infarction (NSTEMI) and unstable angina, in accordance with the guidelines set forth by the European Society of Cardiology.**	HPLC-MS	DCA, CDCA, GCA and GUDCA were positively whilst CA, TDCA, GCDCA, HDCA, UDCA and LCA were negatively assocaited with the risk of MACE. A BA score was associated with an increased risk of MACE in multivariable regression model (HR per 1 SD = 1.35; 95% CI: 1.12, 1.63; p-value = 0.001)	Age, sex, BMI smoking, hypertension, dyslipidemia, T2DM,, unstable angina, acute ST-segment elevation myocardial infarction, non-ST-segment elevation acute myocardial infarction,

	309 patients with ACS			statin medication, beta-blockers, oral antidiabetic medication, insulin medication, diuretics, aspirin, and GFR.
Abbreviations: NASH; non-alcoholic steatohepatitis, NAFLD; non-alcoholic fatty liver disease, BA; Bile acid, LC; liquid chromatography, MS; mass spectrophotometry, 3-DHCA; 3α,7α-dihydroxycholestanoic acid, GCA; glycocholic acid, GCDCA; glycochenodeoxycholic acid, GLCA; glycolithocholic acid, TDCA; taurodeoxycholic acid, TUDCA; tauroursodeoxycholic acid, HPLC; high-performace liquid chromatography, DCA; deoxycholic acid, TCDCA; taurochenodeoxycholic acid, GUDCA; glycoursodeoxycholic acid, MCA; muricholic acid, CDCA; chenodeoxycholic acid, GDCA; glycodeoxycholic acid, TCA; taurocholic acid, T2DM; type 2 diabetes, LDL-C; low-density lipoprotein cholesterol, HDL-C; high-density lipoprotein cholesterol, LCA; lithocholic acid, CDCA-24G; chenodeoxycholic Acid 24-Acyl-β-D-Glucuronide, HDCA; Hyodeoxycholic acid, 12α-OH; 12α-hydroxylated, LCA; lithocholic acid, CA; cholic acid, TLCA taurolithocholic acid.				

1. Bechmann LP, Kocabayoglu P, Sowa JP, et al. Free fatty acids repress small heterodimer partner (SHP) activation and adiponectin counteracts bile acid-induced liver injury in superobese patients with nonalcoholic steatohepatitis. *Hepatology* 2013;57(4):1394-406.
2. Ferslew BC, Xie G, Johnston CK, et al. Altered Bile Acid Metabolome in Patients with Nonalcoholic Steatohepatitis. *Dig Dis Sci* 2015;60(11):3318-28.
3. Jahnel J, Zöhrer E, Alisi A, et al. Serum Bile Acid Levels in Children With Nonalcoholic Fatty Liver Disease. *J Pediatr Gastroenterol Nutr* 2015;61(1):85-90.
4. Na J, Susan SB, Adrian C-R, et al. Suppressed hepatic bile acid signalling despite elevated production of primary and secondary bile acids in NAFLD. *Gut* 2018;67(10):1881.
5. Puri P, Daita K, Joyce A, et al. The presence and severity of nonalcoholic steatohepatitis is associated with specific changes in circulating bile acids. *Hepatology* 2018;67(2):534-48.
6. Chen J, Zheng M, Liu J, et al. Ratio of Conjugated Chenodeoxycholic to Muricholic Acids is Associated with Severity of Nonalcoholic Steatohepatitis. *Obesity (Silver Spring)* 2019;27(12):2055-66.
7. Yara S-i, Ikegami T, Miyazaki T, et al. Circulating bile acid profiles in Japanese patients with NASH. *GastroHep* 2019;1(6):302-10.
8. Caussy C, Hsu C, Singh S, et al. Serum bile acid patterns are associated with the presence of NAFLD in twins, and dose-dependent changes with increase in fibrosis stage in patients with biopsy-proven NAFLD. *Aliment Pharmacol Ther* 2019;49(2):183-93.
9. Zhang Z, Dai W, Weng S, et al. The association of serum total bile acid with non-alcoholic fatty liver disease in Chinese adults: a cross sectional study. *Lipids in health and disease* 2020;19(1):18.
10. Adams LA, Wang Z, Liddle C, et al. Bile acids associate with specific gut microbiota, low-level alcohol consumption and liver fibrosis in patients with non-alcoholic fatty liver disease. *Liver Int* 2020;40(6):1356-65.
11. Sydor S, Best J, Messerschmidt I, et al. Altered Microbiota Diversity and Bile Acid Signaling in Cirrhotic and Noncirrhotic NASH-HCC. *Clin Transl Gastroenterol* 2020;11(3):e00131.
12. Nimer N, Choucair I, Wang Z, et al. Bile acids profile, histopathological indices and genetic variants for non-alcoholic fatty liver disease progression. *Metabolism* 2021;116:154457.
13. Chen T, Zhou K, Sun T, et al. Altered bile acid glycine : taurine ratio in the progression of chronic liver disease. *J Gastroenterol Hepatol* 2022;37(1):208-15.
14. Jung Y, Koo BK, Jang SY, et al. Association between circulating bile acid alterations and nonalcoholic steatohepatitis independent of obesity and diabetes mellitus. *Liver Int* 2021;41(12):2892-902.
15. Wu T, Yang M, Xu H, et al. Serum Bile Acid Profiles Improve Clinical Prediction of Nonalcoholic Fatty Liver in T2DM patients. *Journal of Proteome Research* 2021;20(8):3814-25.
16. Xie G, Jiang R, Wang X, et al. Conjugated secondary 12α-hydroxylated bile acids promote liver fibrogenesis. *EBioMedicine* 2021;66:103290.
17. Kasai Y, Kessoku T, Tanaka K, et al. Association of Serum and Fecal Bile Acid Patterns With Liver Fibrosis in Biopsy-Proven Nonalcoholic Fatty Liver Disease: An Observational Study. *Clin Transl Gastroenterol* 2022;13(7):e00503.
18. Rivera-Andrade A, Petrick JL, Alvarez CS, et al. Circulating bile acid concentrations and non-alcoholic fatty liver disease in Guatemala. *Aliment Pharmacol Ther* 2022;56(2):321-29.
19. Liu AN, Xu CF, Liu YR, et al. Secondary bile acids improve risk prediction for non-invasive identification of mild liver fibrosis in nonalcoholic fatty liver disease. *Aliment Pharmacol Ther* 2023;57(8):872-85.

20. Fitzinger J, Rodriguez-Blanco G, Herrmann M, et al. Gender-Specific Bile Acid Profiles in Non-Alcoholic Fatty Liver Disease. *Nutrients* 2024;16(2).
21. Li W, Shu S, Cheng L, et al. Fasting serum total bile acid level is associated with coronary artery disease, myocardial infarction and severity of coronary lesions. *Atherosclerosis* 2020;292:193-200.
22. Feng X, Zhai G, Yang J, et al. Myocardial Infarction and Coronary Artery Disease in Menopausal Women With Type 2 Diabetes Mellitus Negatively Correlate With Total Serum Bile Acids. *Frontiers in endocrinology* 2021;12:754006.
23. Chong Nguyen C, Duboc D, Rainteau D, et al. Circulating bile acids concentration is predictive of coronary artery disease in human. *Scientific Reports* 2021;11(1):22661.
24. Liu TT, Wang J, Liang Y, et al. The level of serum total bile acid is related to atherosclerotic lesions, prognosis and gut Lactobacillus in acute coronary syndrome patients. *Ann Med* 2023;55(1):2232369.
25. Wen W, Li Q, She J, et al. Predictive value of serum TBA for 2-year MACEs in ACS patients undergoing PCI: a prospective cohort study. *Scientific Reports* 2024;14(1):1733.
26. Mateu-Fabregat J, Mostafa H, Sanchez-Gimenez R, et al. Bile Acids and Risk of Adverse Cardiovascular Events and All-Cause Mortality in Patients with Acute Coronary Syndrome. *Nutrients* 2024;16(7).
